# Xenogeneic Grafts Combined with Platelet Concentrates: Effects on Bone Microarchitecture and Collagen Maturation

**DOI:** 10.3390/dj14100675

**Published:** 2026-10-16

**Authors:** Wanderson Thalles de Souza Braga, Ulli da Costa Cunha Martins, Débora de Souza Ferreira Sávio, Lucia Moitrel Pequeno da Silva, Maria da Conceição Pereira Saraiva, Carlos Fernando Mourão, Roberta Okamoto, Flávia Aparecida Chaves Furlaneto, Michel Reis Messora

**Affiliations:** 1 Department of Oral and Maxillofacial Surgery and Periodontology—DCTBMF, School of Dentistry of Ribeirão Preto, University of São Paulo—USP, Ribeirão Preto 14040-904, SP, Brazil; wanderson.thalles@usp.br (W.T.d.S.B.); ullicosta@usp.br (U.d.C.C.M.); deborasavio@usp.br (D.d.S.F.S.); mdsaraiv@umich.edu (M.d.C.P.S.); flafurlaneto@usp.br (F.A.C.F.); 2 Private Practice, Jaguariúna 13912-696, SP, Brazil; lu.moitrel@hotmail.com; 3 Department of Basic and Clinical Translational Sciences, Tufts University School of Dental Medicine, Boston, MA 02111, USA; carlos.mourao@tufts.edu; 4 Department of Basic Sciences—DCB, School of Dentistry of Araçatuba, Paulista State University—UNESP, Araçatuba 16015-050, SP, Brazil; roberta.okamoto@unesp.br

**Keywords:** bone regeneration, bone substitutes, blood concentrates, tissue engineering

## Abstract

**Background:** This study assessed the impact of xenogeneic bone grafts (XEN), either utilized alone or combined with autologous platelet concentrates (APCs) prepared using three different centrifugation protocols, on the healing of critical-size defects (CSDs) in rat calvaria. **Methods:** Forty rats were assigned to five groups (*n* = 8): Control, XEN, XEN + L-PRF, XEN + A-PRF, and XEN + Bio-PRF. Blood concentrates were prepared by centrifuging blood collected via cardiac puncture: L-PRF (~700 g for 12 min), A-PRF (~208 g for 14 min), and Bio-PRF (~700 g for 8 min). Defects (5 mm diameter) in the right parietal bone of each rat were filled with blood clot, XEN, or XEN combined with L-PRF, A-PRF, or Bio-PRF. Animals were euthanized 60 days post-surgery, and the calvariae were analyzed using histomorphometry and micro-CT. The percentage of mature and immature collagen fibers was analyzed using Picrosirius red staining under polarized light microscopy. **Results:** Groups XEN-A-PRF, XEN-L-PRF, and XEN-Bio-PRF presented higher bone volume (BV/TV) than the control group (14.35 ± 6.42%, 11.96 ± 4.68%, and 14.92 ± 3.47%, respectively, vs. 4.13 ± 2.25%; *p* < 0.05). These groups also presented lower bone porosity (BP) than the control group (*p* < 0.05). The XEN group did not show different values of BV/TV and BP compared to the control group. Only the XEN + A-PRF and XEN + Bio-PRF groups showed a higher percentage of mature collagen fibers (red color) compared to the XEN and control groups (*p* < 0.05). The control and XEN groups showed a higher amount of immature collagen fibers (green color) compared to the XEN + A-PRF, XEN + L-PRF, and XEN + Bio-PRF groups (*p* < 0.05). **Conclusions:** It can be concluded that CSDs treated with XEN combined with APCs presented a more favorable bone microarchitecture than untreated CSDs after a 60-day healing period. Furthermore, CSDs treated with XEN combined with A-PRF or Bio-PRF showed greater collagen maturation than those treated with XEN alone, suggesting that the centrifugation protocol used for preparing APCs may be a decisive factor for the quality of the collagen matrix in newly formed bone tissue.

## 1. Introduction

In recent decades, advancements in bioengineering and cellular and molecular biology have led to a better understanding of tissue regeneration mechanisms [1]. This scientific progress has enabled the development of new biomaterials and methods to enhance the regeneration of specific tissues, thereby driving the concept and development of tissue engineering [1,2].

The use of autologous platelet concentrates (APCs), with particular focus on leukocyte- and platelet-rich fibrin (L-PRF), produced from the centrifugation of the patient’s own blood, has been extensively studied as a low-cost and clinically easy-to-apply autologous biomaterial that enhances tissue regeneration processes, especially when combined with bone scaffolds such as xenogeneic grafts. These concentrates form a three-dimensional matrix containing growth factors, cytokines, inflammatory cells, and platelets, which, when applied to surgical sites, stimulate tissue regeneration [3,4,5,6]. 

L-PRF is a second-generation blood concentrate first described by Choukroun et al. (2001) [7]. It is a three-dimensional fibrin network biological scaffold that provides physical support to various cells, such as neutrophils, macrophages, and fibroblasts. Additionally, it serves as a source of cytokines and growth factors, including platelet-derived growth factor (PDGF), transforming growth factor-beta (TGF-β), insulin-like growth factor (IGF-1), fibroblast growth factor (FGF), vascular endothelial growth factor (VEGF), angiopoietin, and integrin αvβ3 [3,8,9,10]. Consequently, it activates chemotaxis, mesenchymal stem cell differentiation, angiogenesis, and modulation of the immune response. The growth factors released by L-PRF bind to specific tyrosine kinase receptors on the surface of target cells, such as osteoblasts, cementoblasts, and fibroblasts, potentially enhancing tissue regeneration [11,12,13].

It has been demonstrated that changes in centrifugation speeds can alter the distribution and quantity of cells in the blood concentrates [14,15,16,17]. For instance, reducing the speed of centrifugation [14,15] or using horizontal centrifugation increases the concentration of cells trapped in the fibrin networks, which could enhance the regenerative effects of the matrices [16,17,18]. These findings have led to the development of low-speed centrifugation protocols for producing advanced platelet-rich fibrin (A-PRF) [14,15] and platelet-rich fibrin obtained through horizontal centrifugation (Bio-PRF) [16,17,18]. Regarding horizontal centrifugation, recent evidence suggests that it may contribute to improved clinical outcomes when PRF is indicated, due to higher cellular concentrations, more uniform cell distribution within the fibrin matrix, and increased growth factor release compared with fixed-angle centrifugation [19].

Despite several in vitro studies described in the literature, only two in vivo studies [20,21] have demonstrated the impact of different blood concentrate production protocols on bone neoformation. However, these studies assessed the effects of APCs used alone, which complicates the translation of these results to clinical practice, where they are more commonly used in combination with bone grafts for various reconstructive procedures. Therefore, it remains unclear whether differences in blood concentrate preparation protocols can influence the biological response to bone grafts and the quality of newly formed bone. Furthermore, evaluating bone healing based solely on the extent of mineralized tissue may not fully reflect the quality and maturation of the newly formed tissue, since the composition and organization of the organic matrix also contribute to its structural integrity and functional properties [22,23]. In particular, collagen provides the framework for mineral deposition, and alterations in its organization and content have been associated with changes in bone’s mechanical competence [24]. Therefore, assessing collagen maturation may provide complementary information on the quality and ongoing remodeling of newly formed bone, in addition to volumetric assessment alone. Thus, the present study aimed to evaluate the effects of bovine-derived xenogeneic bone grafts (XEN), used either alone or in combination with PRF prepared via three distinct centrifugation protocols, on the healing of critical-size defects (CSDs) created in rat calvaria.

## 2. Materials and Methods

### 2.1. Experimental Materials

#### 2.1.1. Animals

Forty rats (Rattus norvegicus, albinus, Wistar) aged 14 weeks (3.5 months) with body masses of 350 and 450 g were used, obtained after submission and approval of the study protocol by the Committee on Ethics in the Use of Animals (CEUA) at the School of Dentistry of Ribeirão Preto (FORP) of the University of São Paulo (USP), under number 2022.1.244.58.2. All rats were housed in the institutional animal facility and acclimatized for 7 days prior to the experiment. They were kept in groups of three per cage, each lined with sawdust bedding, under controlled conditions including a 12-h light/dark cycle and a temperature maintained between 22 °C and 24 °C, and were fed unrestricted access to a standardized solid diet and water. All animals were healthy and had not undergone any experimental procedures prior to study initiation. No specific criteria for including or excluding animals or data points were established a priori, and all animals that met the general conditions of the study were included.

Sample size was calculated using GraphPad StatMate 2.0 (GraphPad Software, Inc., San Diego, CA, USA) to ensure 80% statistical power with a significance level of 0.05, considering bone volume fraction (BV/TV) as the primary outcome. The calculation was based on an expected mean difference of 4.76 percentage points in BV/TV and a standard deviation of 2.2, using a one-way analysis of variance (ANOVA) for comparison among five groups, with an equal allocation ratio of 1:1:1:1:1. The calculation indicated a minimum sample size of 6 animals per group. Considering an anticipated dropout rate of 20%, the sample size was increased to 8 animals per group, resulting in a total planned sample size of 40 animals [25]. Wistar rats were randomly divided into five groups (*n* = 8): Control, Xenograft (XEN), XEN + L-PRF, XEN + A-PRF, and XEN + Bio-PRF. Randomization was performed using a computer-generated sequence of random numbers prepared in Microsoft Excel by an investigator who was not involved in the subsequent experimental procedures. The operators and outcome examiners were calibrated and blinded to the allocation of animals to the experimental groups. The study coordinator (M.R.M.) was aware of the group allocation during the allocation process and experimental procedures but did not participate in outcome assessment or data analysis.

#### 2.1.2. Blood Collection and PRF Processing

**Figure 1:**
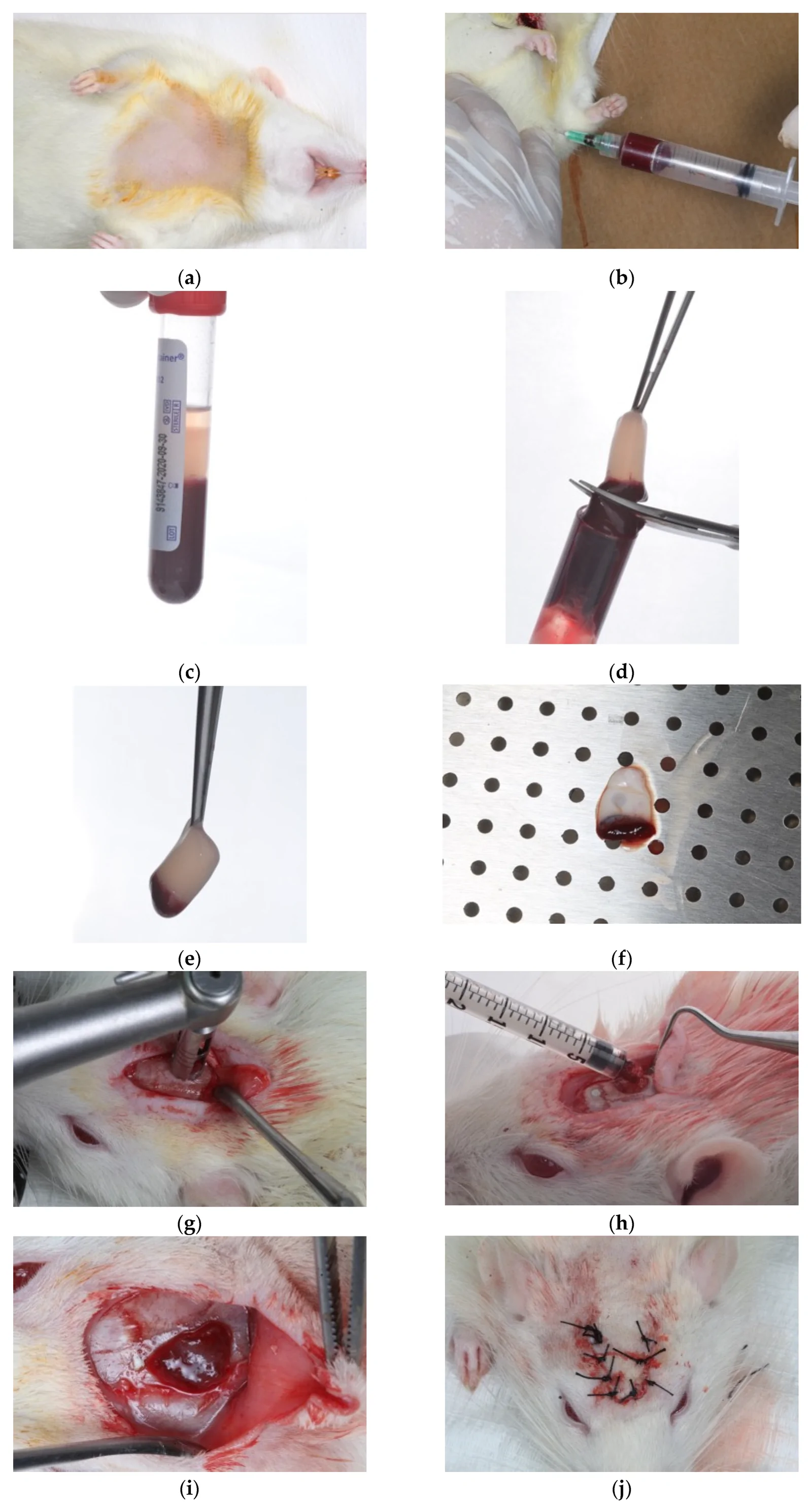
Experimental procedures. (**a**) Shaving and antisepsis of the surgical area; (**b**) blood collection by cardiac puncture; (**c**) tube after centrifugation: observe a horizontal interface between the formed clot and the red cell layer; (**d**) collection of Bio-PRF matrix and separation of the red cell layer using scissors; (**e**) obtained matrix; (**f**) matrix obtained after compression using a specific kit; (**g**) osteotomy with a 5 mm diameter trephine drill; (**h**) filling of the created defect with XEN associated with the blood concentrate; (**i**) placement of a portion of the non-perforated blood concentrate matrix covering the defect; (**j**) flap closure with interrupted simple sutures.

Anesthesia was induced via inhalation of 4% isoflurane (Instituto Biochimico Ind. Farm. Ltda., Itatiaia, Brazil) and maintained at 1.5–3%, followed by intramuscular administration of morphine sulfate (8 mg/kg; Dimorf, Cristália Prod. Quím. Farm. Ltda., Itapira, Brazil) for analgesia. After anesthesia and prior to the creation of the CSD, all animals from all experimental groups (C, XEN, XEN + L-PRF, XEN + A-PRF, and XEN + Bio-PRF) underwent cardiac puncture (Figure 1a,b). 

Approximately 3 mL of blood was collected using 5 mL syringes (Descarpack, São Paulo, Brazil) and transferred to 5 mL silica-coated plastic tubes without additives (BD Vacutainer^®^, Becton, Dickinson and Company, Franklin Lakes, NJ, USA), followed by immediate centrifugation using three distinct protocols for the preparation of APCs. The resulting membranes were visually inspected for consistency and adequate formation before use. In all animals, the PRF membrane was successfully formed at the first blood collection. The volume of blood collected corresponded to the maximum blood volume permitted according to the animal’s body weight. In the XEN + L-PRF group, the blood was processed using the protocol for obtaining L-PRF at 2700 rotations per minute (rpm) for 12 min (~700 g Relative Centrifugal Force [RCFmax]) in an Intra-Spin™ centrifuge (33° rotor angle, 55 mm radius at clot height, 86 mm maximum radius, Intra-Lock^®^ International, Inc., Boca Raton, FL, USA). In the XEN + A-PRF group, blood was processed using the protocol for obtaining A-PRF following the protocol established by Ghanaati et al. (2014) [14]. A-PRF preparation was carried out in the same centrifuge used for L-PRF preparation at 1500 rpm for 14 min (~208 g RCFmax). In the XEN + Bio-PRF group, the blood was processed using the horizontal centrifugation protocol (Bio-PRF) following an adaptation of Miron et al. (2019) [16]. A horizontal centrifuge (Eppendorf 5702 centrifugue; Eppendorf, Hamburg, Germany) was used, and the blood was centrifuged for 8 min at an RCFmax of 700 g. The L-PRF, A-PRF, and Bio-PRF clots were collected with kits and specific instruments (Tissue Regeneration Kit and Xpression™ Box, Intra-Lock^®^ International, Inc., Boca Raton, FL, USA) (Figure 1c–f). 

#### 2.1.3. Creation of Critical Size Defects (CSDs)

The dorsal region of each animal’s skull was shaved, followed by local antisepsis. A semilunar incision was made, and a full-thickness flap was then raised towards the posterior. A 5-mm diameter surgical defect was created in the right parietal bone of each animal using a trephine drill (5 mm Trephine Drill, Neodent^®^, Curitiba, PR, Brazil), mounted on a low-speed contra-angle handpiece set to a constant rotation speed of 1000 rpm, with abundant irrigation using sterile saline solution (Figure 1g). All CSDs were created by the same trained operator to minimize procedural variability.

The defects were filled as follows in each experimental group:

- C group: Filled only with blood clot (n = 8);
- XEN group: Filled only with XEN (Geistlich Bio-Oss^®^ Small [0.25–1 mm], Geistlich Pharma AG, Lucerne, Switzerland) (n = 8);
- XEN + L-PRF group: Filled with XEN combined with L-PRF (n = 8);
- XEN + A-PRF group: Filled with XEN combined with A-PRF (n = 8);
- XEN + Bio-PRF group: Filled with XEN combined with Bio-PRF (n = 8).

The amount of XEN and blood concentrate used in each experimental group was controlled. After defect filling, the surgical sites (Figure 1h,i) were closed with 4-0 silk sutures (Ethicon®, Johnson & Johnson, São José dos Campos, Brazil) to promote primary wound healing (Figure 1j). Postoperative care included intramuscular Penicillin G-benzathine (Pentabiótico Veterinário Pequeno Porte, Fort Dodge Animal Health^®^, Campinas, SP, Brazil; 24,000 IU/kg) administered immediately after surgery and every 48 h for one week. Pain management was provided with daily intramuscular injections of Tramadol hydrochloride 8 mg/kg (Cronidor 2%, Agener União^®^, Apucarana, PR, Brazil) and Meloxicam 0.2 mg/kg for two days. Animals were monitored daily for any adverse clinical signs, such as respiratory distress, lethargy, abnormal posture, inability to access food or water, and wounds. No adverse effects were observed during the experiment. No animals were excluded due to postoperative complications or experimental failures.

#### 2.1.4. Euthanasia

Sixty days after the creation of the CSDs, the animals were euthanized using inhaled isoflurane at a concentration of 4–5%. Calvarial specimens, encompassing the original defect site and adjacent tissues, were excised in blocks and fixed in 10% neutral buffered formalin for 24 h.

### 2.2. Experimental Methods

#### 2.2.1. Analysis by Micro-Computed Tomography (Micro-CT)

Calvarial samples were scanned using cone-beam micro-computed tomography (micro-CT) with a SkyScan 1172 system (SkyScan N.V., Kontich, Belgium), operating at a high spatial resolution of 10 micrometers (µm). The X-ray source was set to an acceleration voltage of 60 kV and a current of 165 µA to optimize image quality and resolution. Using DataViewer version 1.4.3 software (SkyScan N.V., Kontich, Belgium), three-dimensional (3D) images were reconstructed from rotational scans of the samples.

A precise region of interest (ROI) was established with a diameter of 5 mm, corresponding exactly to the location of the original CSD. Within this ROI, a volume of interest (VOI) measuring 0.5 mm × 5 mm × 5 mm was defined, based on previous methodologies [20,21], to focus the quantitative analysis on the defect area and its immediate surroundings. A grayscale range of 0–255, with a threshold between 80 (minimum) and 170 (maximum), was used to evaluate the trabecular bone tissue present in the calvaria within each VOI. The same threshold values were applied to all specimens.

A trained, calibrated, and blinded examiner performed the reconstruction and segmentation and conducted a detailed assessment of the bone microarchitecture within each VOI using CT-Analyzer^®^ software (version 1.13.5.1+, Bruker, Kontich, Belgium). The evaluation included several key structural parameters of each VOI: (i) the percentage of the VOI occupied by bone tissue (BV/TV); (ii) the percentage of the VOI occupied by bovine bone graft—remaining particles volume (RPV); (iii) the percentage of bone porosity within the VOI (BP); (iv) the number of trabecular bones present in the VOI (Tb.N); (v) the separation of trabeculae in the VOI (Tb.Sp); and (vi) the connectivity density between the trabecular structures present in the VOI (Conn.Dn).

#### 2.2.2. Histological Analysis

After micro-CT scanning, the specimens fixed in formalin and stored in 70% ethanol were washed in running water and subsequently decalcified in a 4% ethylenediaminetetraacetic acid (EDTA) solution for 8 weeks. Following the decalcification process, each specimen was longitudinally divided along the center of the original defect into two parts (A and B), using reference markings filled with amalgam [20,21]. The specimens were then processed and embedded in paraffin. Serial longitudinal sections of 4 µm thickness were cut from the center of the original surgical defect. Two sections from each animal were stained using hematoxylin and eosin (HE) staining, and another two sections were stained with Picrosirius red. These sections were then subjected to photomicrography using a light microscope. For each histological parameter, the values obtained from the two sections were averaged, and the resulting mean value for each animal was used as the unit of analysis in the statistical analyses.

#### 2.2.3. Histomorphometric Analysis

- Hematoxylin and eosin (HE) staining sections

The histomorphometric analysis was conducted using a computer-based image assessment system and specific image acquisition and analysis software (LAS EZ v. 4.1.0, Leica Microsystems^®,^ Wetzlar, Germany). For each selected histological section, photomicrography was performed using a trinocular light and fluorescence microscope (model DMLB, Leica Microsystems GmbH, Wetzlar, Heidelberg, Germany) with a 1.6× objective, coupled with a camera (DFC300FX, Leica Microsystems GmbH, Wetzlar, Heidelberg, Germany).

In each captured image, the analysis area corresponding to the center of the CSD was defined as the total area (TA). Within each TA, the new bone area (NBA) and the remaining particles area (RPA) were selected and defined. The TA value was considered as 100% of the analyzed area, and the NBA value was calculated as a percentage of the TA.

Each section was also qualitatively evaluated for the inflammatory profile present and the histopathological characteristics of the newly formed bone, using 10× and 20× objectives.

- Picrosirius red staining sections

**Figure 2:**
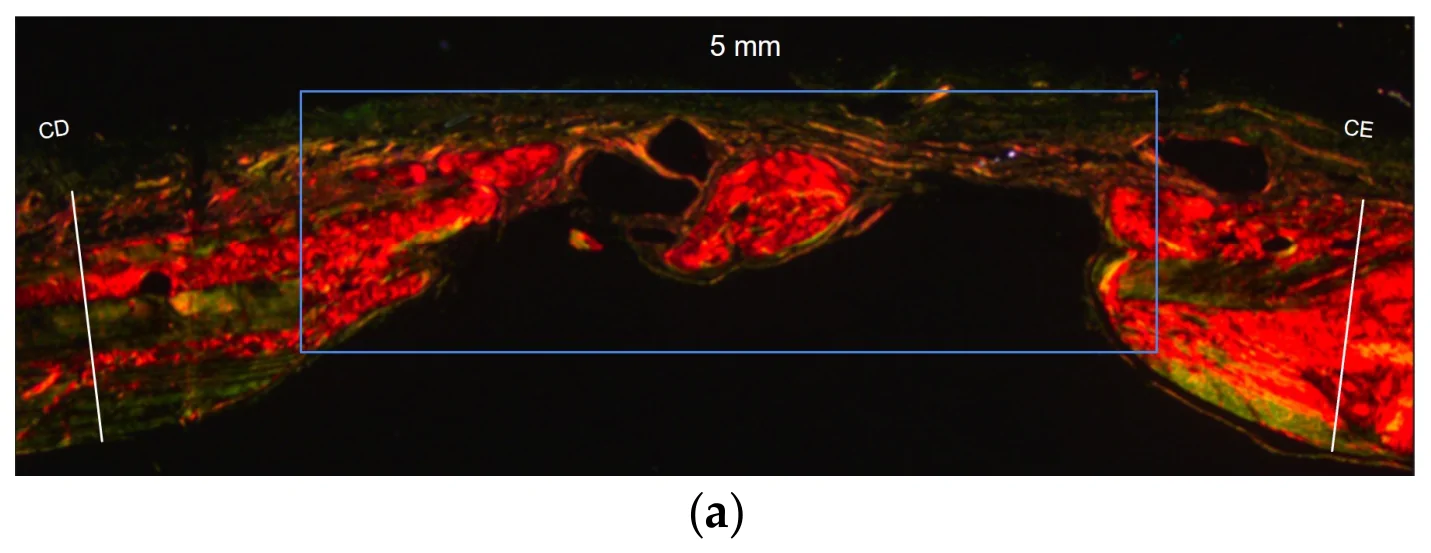

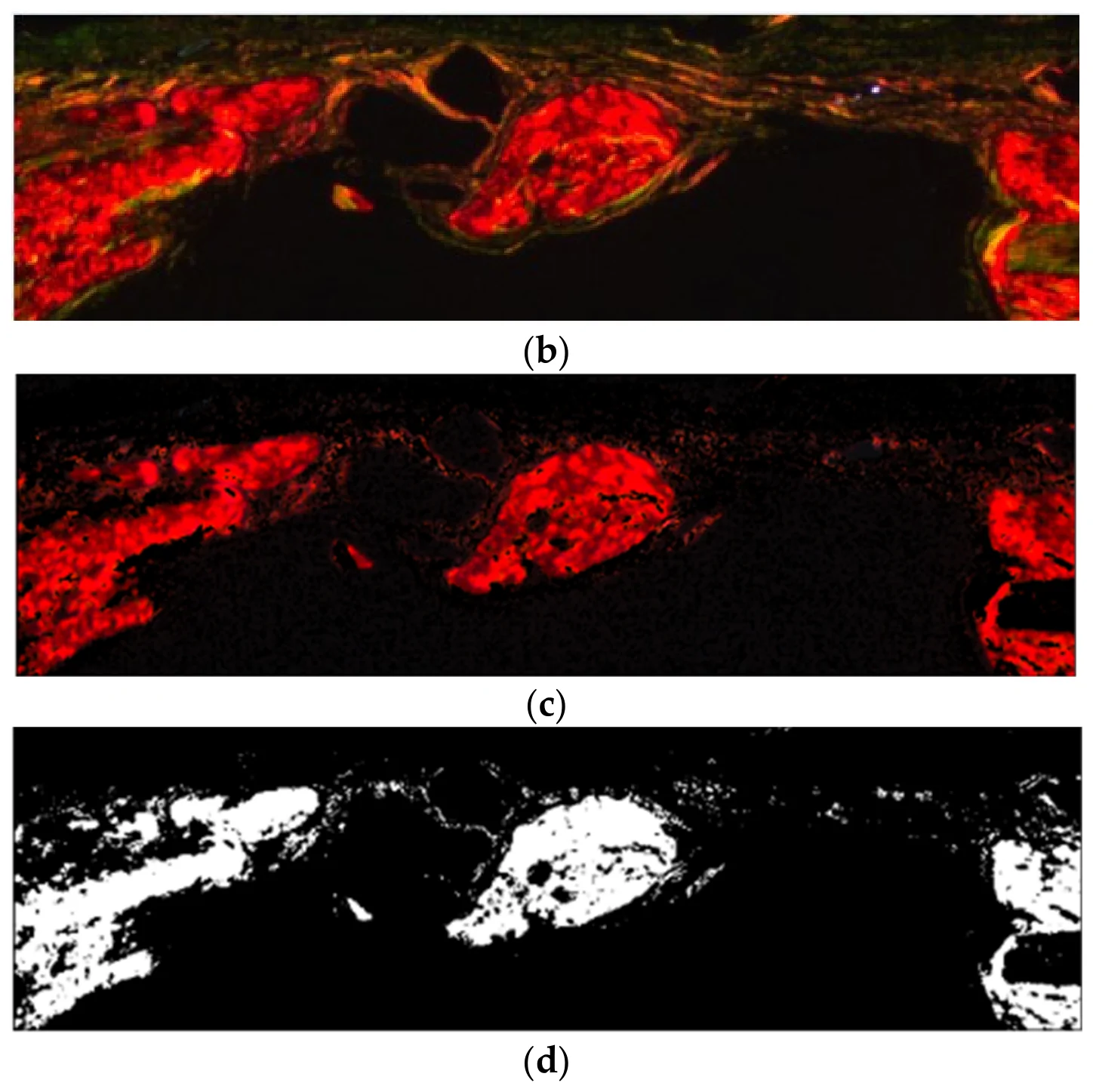
Photomicrography of histological sections for collagen type I deposition analysis. (**a**) The total area (TA) is outlined by the blue line and corresponds to the area of the calvaria where the surgical defect was originally created. The height of the TA corresponds to the average of the height of the right stump (RS) and the left stump (LS). The width of the TA is 5 mm, corresponding to the width of the original defect. Magnification = 1.6×; (**b**) Crop of the TA to be analyzed; (**c**) image with only the red tones selected by the ImageJ software; (**d**) marking after automatic threshold selection for counting the percentage of area selected in the color to be analyzed.

The sections were captured using a trinocular microscope for brightfield and fluorescence (model DMLB, Leica Microsystems GmbH, Wetzlar, Heidelberg, Germany) under polarized light, with a 1.6× objective. Using ImageJ software version 1.43g (National Institutes of Health, Bethesda, MD, USA), the analysis area corresponding to the center of the defect was delineated, termed the TA. For intra-observer calibration, one-third of the sample was evaluated twice, with a 48-h interval between assessments. The intraclass correlation coefficient (ICC) was used to determine the reproducibility of the evaluations. The ICC was 0.984 (95% confidence interval [CI]: 0.939–0.996), indicating excellent reproducibility. After determining the TA, the images were subjected to channel selection for red or green, with all other colors being subtracted from the region to analyze the percentage of type I or mature collagen fibers (red color) and type III or immature collagen fibers (green color). The thresholds for color segmentation were automatically determined by the software. The value of TA was considered to be 100% of the analyzed area, and the red- or green colored area was calculated as a percentage of TA (Figure 2a–d). It is important to note that any collagen fibers within the defect area that were not part of the trabecular bone, as well as soft tissues and remaining biomaterial particles, were excluded from consideration.

### 2.3. Statistical Analysis

Statistical analysis was performed using GraphPad Prism software (GraphPad Software, Inc., v. 5.01, San Diego, CA, USA), with a significance level set at 5% (*p* < 0.05). The animal was considered the statistical unit. No animals or data points were excluded from any experimental group or analysis. Data were grouped and presented as means with standard deviations. The normality of data distribution was assessed separately for each outcome variable using the Shapiro–Wilk test. The significance of differences between groups for all variables was determined by ANOVA, followed by the Tukey post hoc test. Effect sizes were calculated using Cohen’s d for pairwise comparisons.

## 3. Results

### 3.1. Analysis by Micro-Computed Tomography (Micro-CT)

**Figure 3:**
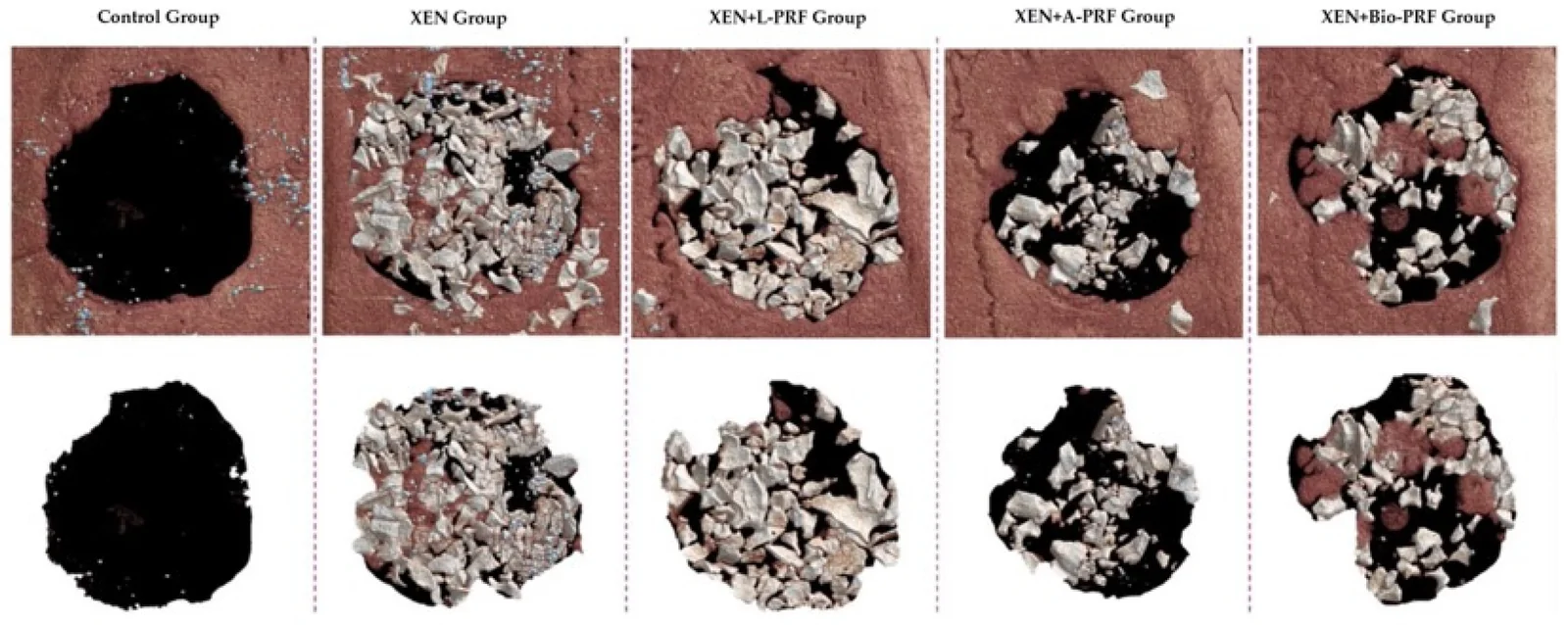
Rendered reconstructions of the Control, XEN, XEN + L-PRF, XEN + A-PRF, and XEN + Bio-PRF.

The three-dimensional reconstructions of the calvarial samples are presented in Figure 3. The mean values and standard deviations for BV/TV, RPV, BP, Tb.N, Tb.Sp and Conn.Dn across all experimental groups, along with the results of intergroup comparisons, are displayed in Table 1. The XEN + A-PRF, XEN + Bio-PRF, and XEN + L-PRF groups showed significantly higher BV/TV values compared to the C group (mean difference (MD) = −7.8, *p* = 0.032, Cohen’s d = −1.71; MD = −10.78, *p* = 0.003, d = −2.36; MD = −10.22, *p* = 0.002, d = −2.23, respectively), as well as higher Tb.N (MD = −1.35, *p* = 0.002, d = −2.17; MD = −1.66, *p* < 0.001, d = −2.68; MD = −1.62, *p* < 0.001, d = −2.61, respectively), higher Conn.Dn (MD = −0.0001, *p* = 0.019, d = −1.64; MD = −0.00018, *p* < 0.001, d = −2.88; MD = −0.00017, *p* < 0.001, d = −2.74, respectively), lower BP (MD = 9.5, *p* = 0.027, d = 1.85; MD = 12.62, *p* = 0.001, d = 2.46; MD = 10.22, *p* = 0.008, d = 1.99, respectively), and lower Tb.Sp (MD = 0.09, *p* = 0.008, d = 1.82; MD = 0.14, *p* < 0.001, d = 2.72; MD = 0.13, *p* < 0.001, d = 2.6, respectively). The XEN group did not differ significantly from the C group in terms of BV/TV (MD = −5.14, *p* = 0.281, d = −1.12) and BP (MD = 9.32, *p* = 0.076, d = 1.82); however, it demonstrated significantly higher Tb.N (MD = −1.39, *p* = 0.003, d = −2.24) and Conn.Dn (MD = −0.00013, *p* = 0.002, d = −2.03) values, and lower Tb.Sp (MD = 0.15, *p* < 0.001, d = 2.95) values. The XEN + Bio-PRF group showed a trend toward higher Conn.Dn compared to the XEN + A-PRF group (MD = −0.000078, *p* = 0.084, d = −1.24). No statistically significant differences were found among the XEN, XEN + A-PRF, XEN + Bio-PRF, and XEN + L-PRF groups regarding RPV or any of the other evaluated parameters (all *p* > 0.05).

### 3.2. Histomorphometric Analysis

#### 3.2.1. Histopathological Analysis

**Figure 4:**
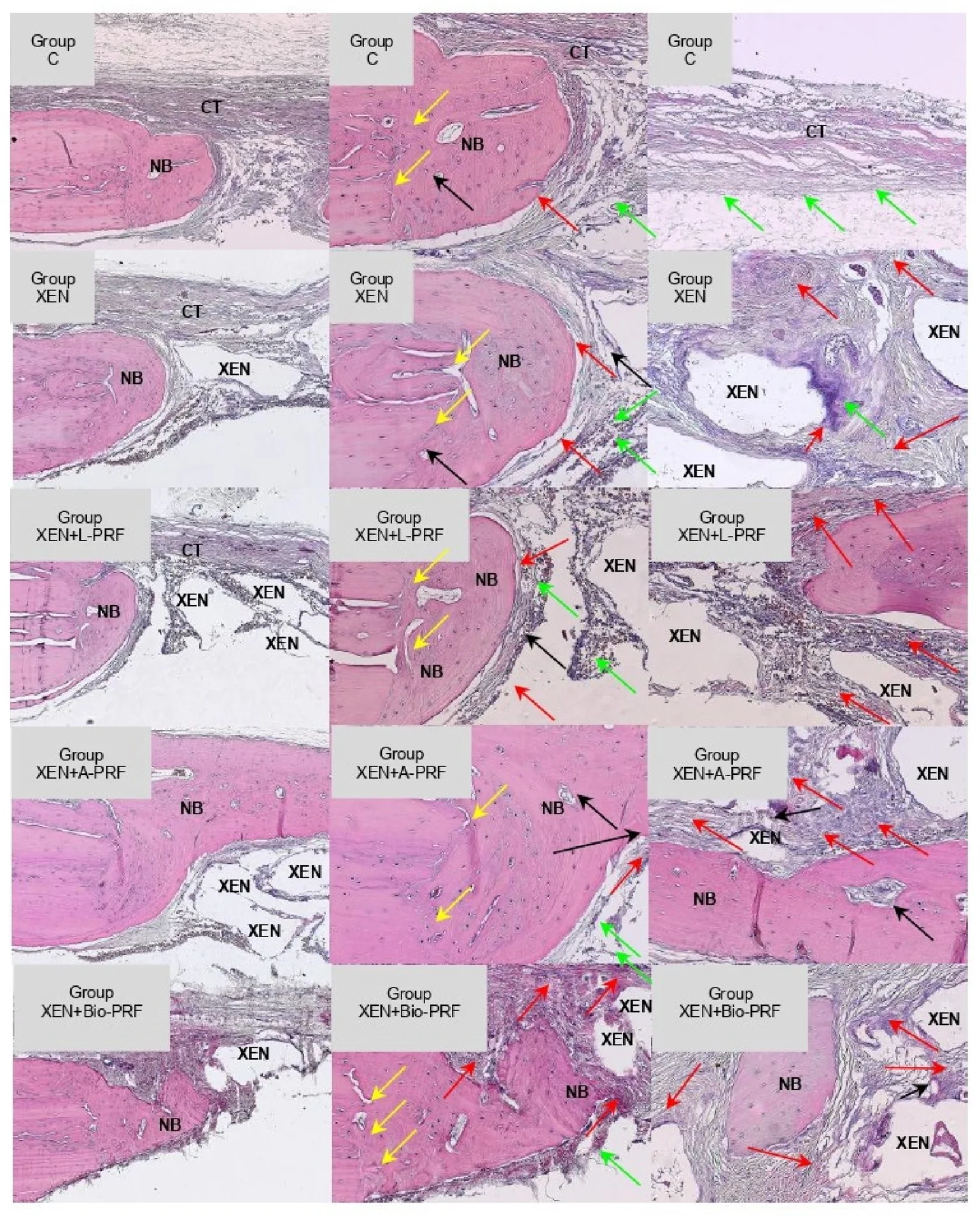
Representative images of histological sections from the area near the original bone margins of the defects in groups C, XEN, XEN + L-PRF, XEN + A-PRF, and XEN + Bio-PRF. NB = newly formed bone; XEN = residual xenograft particle; CT = connective tissue; red arrow = osteoid matrix; black arrow = blood vessels; yellow arrow = margins of the surgically created defect; green arrow = area of inflammatory infiltrate. Images in the right column show regions distant from the surgical defect margin. Staining: hematoxylin and eosin. Original magnification = 10× (left column); 20× (middle and right columns).

Representative histological images of the area adjacent to the original bone margins of the defects in all experimental groups are shown in Figure 4.

- Control Group

At the defect margins, a limited amount of newly formed bone was detected, accompanied by osteoblasts positioned around the nascent bone tissue. Most of the defect area was filled with connective tissue rich in collagen fibers aligned parallel to the wound surface. This tissue appeared thinner than the original bone at the defect edges. In some samples, marked bone resorption was evident at the borders. Across all specimens, blood vessels and fibroblasts were sparsely observed. Moreover, an inflammatory infiltrate composed of lymphocytes and plasma cells was present throughout the defect site.

- XEN Group

Newly formed bone tissue containing osteocytes was observed near the edges of the surgical defect, as well as surrounding and interspersed among the remaining bone graft particles. Collagen fibers within the defect area appeared more organized compared to those in the control group. Most of the residual graft particles showed advanced resorption, associated with a prominent vascular network and an evident osteoid matrix. A mild inflammatory infiltrate was also present.

- XEN + L-PRF Group

At the margins of the defects, areas of bone resorption appeared less prominent compared to the XEN and C groups, and osteoblasts were readily observed. In most samples, the newly formed bone tissue contained numerous osteocytes and blood vessels, as well as distinct islands of new bone and residual particles located in the central regions of the defect. Collagen fibers surrounding the defect and residual particles exhibited a more organized arrangement than those seen in the C and XEN groups. Similar to the XEN group, an osteoid matrix was present around the remaining xenograft particles. Notably, this group showed little residual biomaterial throughout the defect. Additionally, blood vessels and newly formed bone were observed throughout the defect area.

- XEN + A-PRF Group

Similar to the XEN + L-PRF group, the defect borders in this group showed prominent osteoblasts and less evident areas of bone resorption compared to the C and XEN groups. The newly formed bone tissue exhibited features comparable to those observed in the XEN + L-PRF group, including numerous osteocytes and prominent vascularization. In some specimens, continuous bone formation extending from the defect margins toward the central region was noted. As seen in the XEN and XEN + L-PRF groups, an osteoid matrix surrounded the residual graft particles. Blood vessels and newly formed bone were observed along the defect length. The remaining biomaterial particles were less prominent than in the XEN group but more evident than in the XEN + L-PRF and XEN + Bio-PRF groups. A mild inflammatory infiltrate was also observed.

- XEN + Bio-PRF Group

As observed in the XEN + L-PRF and XEN + A-PRF groups, the defect borders in this group showed prominent osteoblasts and less evident areas of bone resorption compared to the C and XEN groups. The newly formed bone tissue displayed a prominent osteoid matrix and rich vascularization, consistent with findings in the XEN + L-PRF and XEN + A-PRF groups. In some specimens, continuous bone formation was evident from the margins toward the center of the defect. Like the XEN, XEN + L-PRF, and XEN + A-PRF groups, an osteoid matrix was present around residual graft particles. The remaining biomaterial was less prominent than in the XEN and XEN + A-PRF groups. Additionally, blood vessels were readily observed throughout the defect. A mild inflammatory infiltrate was also present.

#### 3.2.2. Histometric Analysis of HE-Stained Sections

The means and standard deviations of NBA and RPA, as well as the results of the intergroup comparisons, are shown in Table 2. There were no statistically significant differences in NBA and RPA values between the experimental groups.

**Table 2:** New bone area (NBA) and residual particles area (RPA) in groups C, XEN, XEN + L-PRF, XEN + A-PRF, and XEN + Bio-PRF.

Variable	C (Mean ± SD)	XEN (Mean ± SD)	XEN + L-PRF (Mean ± SD)	XEN + A-PRF (Mean ± SD)	XEN + Bio-PRF (Mean ± SD)	*p*-Value
NBA	0.07 ± 0.06 ^a^	0.15 ± 0.22 ^a^	0.2 ± 0.32 ^a^	0.28 ± 0.31 ^a^	0.22 ± 0.22 ^a^	>0.05
RPA	-	0.54 ± 0.43 ^a^	0.44 ± 0.28 ^a^	0.64 ± 0.42 ^a^	0.59 ± 0.18 ^a^	>0.05

Data are presented as mean ± standard deviation (SD). Different superscript letters indicate significant differences between groups according to Tukey’s post hoc test. The *p*-value represents the overall between-group comparison by one-way ANOVA (*p* < 0.05).

#### 3.2.3. Quantitative Analysis of Collagen with Red or Green Birefringence

By polarizing the collagen fibers stained with Picrosirius red, it was possible to assess the quality of the collagen in the bone matrix through birefringence and the organization of type I collagen fibers (more organized—reddish color—more mature fibers) and type III collagen fibers (disorganized—greenish color—immature fibers) [25,26,27]. The means and standard deviations of the red area (%) and green area (%), as well as the results of the intergroup comparisons, are depicted in Table 3.

Regarding the quantity of mature collagen fibers, only the XEN + Bio-PRF and XEN + A-PRF groups showed significantly higher values compared to the C (MD = −4.23, *p* < 0.001, d = −2.97; MD = −3.57, *p* < 0.001, d = −2.51, respectively) and XEN groups (MD = −4.16, *p* < 0.001, d = −2.92; MD = −3.5, *p* = 0.002, d = −2.46, respectively). In terms of immature collagen fibers, the C and XEN groups exhibited significantly higher values compared to the XEN + L-PRF (MD = 5.52, *p* = 0.002, d = 2.31; MD = 6.4, *p* < 0.001, d = 2.68, respectively), XEN + A-PRF (MD = 8.12, *p* < 0.001, d = 3.4; MD = 9, *p* < 0.001, d = 3.76), and XEN + Bio-PRF groups (MD = 7.32, *p* < 0.001, d = 3.06; MD = 8.2, *p* < 0.001, d = 3.43). 

## 4. Discussion

This in vivo study investigated the effects of XEN, used alone or in combination with APCs prepared through three distinct centrifugation protocols (L-PRF, A-PRF, and Bio-PRF), on the healing of CSDs in rat calvaria. While micro-CT and histomorphometric analyses revealed no statistically significant differences in the total volume of newly formed bone among the XEN and XEN + APC groups at 60 days post-operation, notable differences were observed in the collagen composition of the regenerated bone tissue. These variations were directly influenced by the specific centrifugation protocol used to produce the APCs.

Understanding and identifying the cellular and molecular mechanisms involved in tissue regeneration is essential for the development of surgical techniques and biomaterials [26,27]. For regeneration to occur, there must be interactions between three main pillars of tissue engineering; one of which is the scaffold, like the extracellular matrix, that remains structurally stable after the overlay of soft tissues. An ideal scaffold should improve cellular viability, adhesion, proliferation, cell homing, osteogenic differentiation, and vascularization. Moreover, it should avoid host immune responses, interact with surrounding tissues, maintain three-dimensional stability, and be biologically active [28]. Bio-Oss^®^ was used in the present study due to its exceptional osteoconductive properties [29]. This biomaterial is widely recognized as a slow-resorbing xenograft that promotes blood vessel proliferation and the migration of bone cells onto its particles [30]. However, Bio-Oss does not present osteoinductive potential nor osteogenic properties. The aim in the present study was to combine it with APCs to enhance its properties during bone regeneration processes. APCs provide a slow and gradual release of cytokines and growth factors during a critical period for collagen, fibronectin, and other extracellular matrix component formation that guides tissue regeneration [8,10,13]. This gradient of cytokines can primarily attract mesenchymal stem cells (MSCs) from bone marrow and those in regions adjacent to the wound or defect, followed by their differentiation into osteoprogenitor cells.

L-PRF, a second-generation blood concentrate, is a biomaterial obtained through the centrifugation of autogenous blood [6,7,8,11,17]. Its use in the bone regeneration of CSDs in rats has been extensively studied over the past 10 years and has shown satisfactory results in bone neoformation [20,21,25,31,32,33,34,35]. The modification of the protocol initially established by Choukroun and colleagues (2001) [7], focusing on changes in RCF, centrifugation time, and the introduction of new centrifugation devices, has led to the development and emergence of new protocols, such as A-PRF [14] and Bio-PRF [16]. These new matrices produced from modified protocols have demonstrated various advantages over the original matrices in various in vitro and in vivo evaluations [14,16,18,36,37]. Indeed, the scientific literature increasingly aims to obtain a blood concentrate matrix with higher mechanical resistance, prolonged degradation time, and greater retention and release rates of growth factors.

Few in vivo studies have directly compared the impact of different protocols for blood concentrate preparation on bone regeneration. Using microtomographic and histomorphometric evaluations, Silva et al. (2022) [20] demonstrated that both L-PRF and A-PRF enhanced bone formation in CSDs in rats at 30 days post-surgery. Although no statistically significant differences in the volume of newly formed bone were found between the L-PRF and A-PRF groups, the authors emphasized key findings from qualitative histological assessments and microarchitectural analyses, which revealed distinct characteristics in bone tissue organization and quality among the experimental groups. According to the authors, compared to defects filled with L-PRF, defects treated with A-PRF showed a greater amount of osteoid matrix and a smaller spacing between bone trabeculae. The authors also argue that studies with longer follow-up periods and immunohistochemical analyses could better explain the obtained results and highlight possible molecular impacts resulting from changes in PRF matrix composition. Sávio and colleagues (2023) [21] demonstrated through microtomographic, histomorphometric and fluorescence analyses that blood concentrate matrices produced from fixed-angle centrifuges (L-PRF and A-PRF) and horizontal centrifugation (H-PRF; Bio-PRF) enhanced bone neoformation in CSDs in rat calvaria in evaluations performed at 30 days post-operatively. It was also observed that CSDs treated with Bio-PRF showed higher values of BV/TV, Tb.N, and alizarin precipitation compared to those treated with A-PRF and L-PRF. The authors concluded that Bio-PRF has greater biological potential in the bone neoformation process.

Previous studies evaluating different protocols for preparing autologous platelet concentrates in bone regeneration have primarily focused on short-term healing periods and have generally investigated APCs without the concomitant use of bone graft materials. In contrast, to the best of our knowledge, the present study is the first to evaluate the combined use of XEN with three distinct APC preparation protocols (L-PRF, A-PRF, and Bio-PRF) in CSDs of rat calvaria during a late-stage healing period. Histomorphometric analysis revealed no significant differences in NBA among the XEN and XEN + APC groups, indicating that the addition of APCs did not result in detectable differences in the amount of newly formed bone at 60 days. This absence of differences may be related, at least in part, to the relatively late healing period evaluated in the present study, as differences associated with the early kinetics of bone formation may become less evident as the healing process progresses. This interpretation is consistent with previous findings showing differences between XEN and XEN + L-PRF at 30 days, but not at 60 days [31], as well as greater bone volume following XEN + L-PRF compared with XEN alone when a shorter healing period was evaluated [25]. Interestingly, the histomorphometric and micro-CT findings did not show complete agreement in our study. While histomorphometric analysis showed no significant differences among the experimental groups, including the control group, micro-CT analysis revealed a consistent numerical pattern of more favorable microarchitectural parameters in the XEN + APC groups, although direct comparisons with XEN alone did not reach statistical significance. This apparent discrepancy may be explained by the distinct nature and sampling characteristics of the two methods. Histomorphometric analysis evaluates the area of newly formed bone within histological sections, and in the present model, was based on a relatively limited central region of the defect. In contrast, micro-CT provides a three-dimensional assessment of bone volume and microarchitecture throughout the entire defect, potentially capturing spatial variations in mineralized tissue that may not be represented in the histological section. Therefore, the complementary nature of these techniques should be considered when interpreting the absence of significant differences in NBA alongside the microarchitectural patterns observed by micro-CT.

It is also important to highlight that tissue volume formation does not equate to tissue maturation, as the treated defect may be filled with immature tissue, compromising its functional viability [27]. The quality of bone tissue is not limited to its mineralization but also includes the quantity and quality of collagen fibers, which are both crucial for bone matrix mineralization and thus for the mechanical quality of bone tissue [22,23]. In the Picrosirius red analysis, the more organized (mature) fibers appear redder, while the more disorganized (immature) fibers exhibit a greener coloration [23,38,39]. Type I collagen, representing more mature fibers, acts as a support for bone matrix mineralization, directly influencing the microarchitecture of trabecular bone [40]. In this study, it was observed that only the XEN + A-PRF and XEN + Bio-PRF groups had a higher amount of mature collagen fibers (type I) compared to the XEN group. This finding may be related to differences in the cellular composition, fibrin architecture, and growth factor release kinetics generated by the centrifugation protocols used to prepare A-PRF and Bio-PRF. Although these mechanisms were not directly evaluated in the present study, low-speed centrifugation has been shown to promote a greater retention of platelets and leukocytes within the PRF clot, rather than their displacement toward the buffy coat or red blood cell layer, potentially resulting in a more prolonged and enhanced release of growth factors [14,15]. Moreover, horizontal centrifugation may further increase the concentration of cellular components in PRF compared with fixed-angle centrifugation by promoting more efficient cell separation and reducing mechanical stress [16]. These differences may influence the organization and maturation of the collagen-rich extracellular matrix, and consequently, the quality of the newly formed tissue, even in the absence of a significant increase in the overall bone volume. Thus, certain protocols developed with advanced centrifugation approaches can potentially enhance the osteoconductive properties of XEN and that the type of preparation protocol used can provide different organic matrix compositions in newly formed bone tissue. 

Considering the effects of this organic matrix on the functionality and strength of bone tissue [39], and since poorer collagen fiber organization and reduced collagen content may be associated with impaired bone matrix strength [24], the greater collagen organization observed in the XEN + A-PRF and XEN + Bio-PRF groups may indicate differences in the quality and maturation of the newly formed tissue. Although the micro-CT analysis did not reveal differences in bone microarchitectural parameters between the XEN and PRF-containing groups, the observed differences in collagen organization may reflect qualitative aspects of the regenerated tissue that are not captured by conventional microtomographic measurements. This distinction may be relevant because bone quality and microarchitecture can influence the subsequent behavior of bone under functional loading. In a recent clinical study, Dong et al. (2026) [41] demonstrated that peri-implant bone microstructure was associated with long-term marginal bone loss, with a higher BV/TV associated with less marginal bone loss and greater Tb.Sp associated with increased marginal bone loss over 3–8 years of functional loading. The authors further discussed that bone microarchitecture may influence the dissipation and transmission of mechanical stresses, and consequently, the remodeling response of peri-implant bone. Although these findings cannot be directly extrapolated to the present experimental model, they support the concept that differences in bone quality may have implications for subsequent remodeling behavior. Thus, the greater collagen organization observed in the XEN + A-PRF and XEN + Bio-PRF groups may represent a potentially relevant qualitative feature of the regenerated tissue, despite the absence of differences in micro-CT parameters. Although mechanical properties and subsequent remodeling around implants were not evaluated in the present study, these findings may be of potential clinical interest for procedures involving bone regeneration and implant site preparation, such as guided bone regeneration and ridge augmentation. Therefore, future studies are important to, for example, longitudinally evaluate the degree of bone remodeling associated with dental implants installed in bone tissue regenerated with XEN combined with blood concentrates produced by different protocols.

In this study, despite differences in collagen maturation among the groups, neither micro-CT analysis of RPV nor histomorphometric analysis of RPA revealed significant differences in the amount of remaining graft particles between the XEN and XEN + APC groups. These findings suggest that APC-associated differences in collagen matrix maturation were not accompanied by detectable differences in graft particle resorption at the evaluated time point, indicating that graft resorption was not substantially influenced by the observed differences in matrix quality. Similarly, a previous preclinical study in ovariectomized rats with surgically created calvarial defects reported no differences in residual graft particles between the XEN and XEN + L-PRF groups after 30 days, as assessed by both micro-CT and histomorphometric analyses [25].

In the present study, XEN + A-PRF and XEN + Bio-PRF showed a more favorable collagen maturation profile than XEN + L-PRF, with greater proportions of mature collagen. Notably, the comparison between XEN + A-PRF and XEN + Bio-PRF also showed a near-significant difference in Conn.Dn on micro-CT, with numerically higher values for XEN + Bio-PRF. These findings raise the possibility that differences in PRF preparation protocols may influence specific characteristics of the regenerative response. In a recent study [19], the outcomes of comparing the centrifugation devices were investigated. The overview review article offered deeper insights into the advantages of H-PRF when compared to fixed-angle methods across a wide range of regenerative medical and dental applications. A total of 75 studies were included, with 13 studies directly comparing horizontal centrifugation to fixed-angle centrifugation for producing PRF, while the remaining 62 studies were non-comparative and focused on expanding the uses and clinical applications of H-PRF. These studies span categories such as cell concentrations, fibrin matrix structure, growth factor release, antibacterial and anti-inflammatory properties, and regenerative applications in bone, periodontal, cartilage, skin, hair, regenerative endodontics, corneal defect repair, wound healing, and soft tissue regeneration. Of the studies comparing horizontal to fixed-angle centrifugation, 84.6% favored horizontal centrifugation, while 15.4% found no difference. None of the studies favored fixed-angle centrifugation. Nevertheless, further comparative clinical studies are warranted to further support these findings [19].

Lastly, it has also been reported that the PRF tubes utilized during the production of PRF likely matter even more so than the centrifugation device itself [42]. It has been reported that many tube types contain chemical additives or other substances that may either delay or promote or delay the clotting process and the strength of the clot [43]. In the present animal study, the standard 10 mL PRF tubes from each manufacturer could not be utilized owing to the small size of the animals, and instead, 3 mL of blood was drawn into 5 mL tubes without chemical additives. Therefore, it remains to be clinically observed what effect the tube types from each of the respective manufacturers will have on the final PRF outcomes, including their subsequent regenerative potential.

In conclusion, this study demonstrated that the centrifugation protocol used for the preparation of APCs was shown to impact the quality of newly formed bone tissue when combined with XEN. Some additional limitations of this study should be considered when analyzing the results. Since the bone defects were not protected by a membrane (barrier), it is not possible to evaluate whether the results would be the same under standard guided bone regeneration principles/conditions. The levels of growth factors in the blood concentrates were also not measured in the smaller tube sizes utilized, so a direct correlation between the levels and types of factors present and the obtained bone formation could not be evaluated/estimated. Future studies should address this topic for a better understanding of biological response variability. The use of animals with systemic impairments and on a larger phylogenetic scale should also be considered in future studies to better elucidate the role of APCs in promoting faster bone formation and their optimal benefit in clinical practice.

## 5. Conclusions

Within the limits of this study, it can be concluded that defects treated with XEN combined with A-PRF or Bio-PRF, when evaluated at a later stage of healing, show neoformed bone tissue with greater collagen maturation compared to those treated with XEN alone. Additionally, the type of protocol used for preparing blood concentrates appears to be crucial for the quality of the collagen matrix in the neoformed bone tissue.

## Figures and Tables

**Table 1:** Bone volume (BV/TV), bone porosity (BP), trabecular separation (Tb.Sp), number of trabeculae (Tb.N), connectivity density (Conn.Dn), and remaining particles volume (RPV) in groups C, XEN, XEN + L-PRF, XEN + A-PRF, and XEN + Bio-PRF.

Variable	C (Mean ± SD)	XEN (Mean ± SD)	XEN + L-PRF (Mean ± SD)	XEN + A-PRF (Mean ± SD)	XEN + Bio-PRF (Mean ± SD)	*p*-Value
BV/TV (%)	4.13 ± 2.25 ^a^	9.27 ± 3.42 ^ab^	14.35 ± 6.42 ^b^	11.96 ± 4.68 ^b^	14.92 ± 3.47 ^b^	<0.05
BP (%)	95.87 ± 2.25 ^a^	86.55 ± 7.59 ^ab^	85.65 ± 6.42 ^b^	86.37 ± 2.6 ^b^	83.247 ± 4.79 ^b^	<0.05
Tb.Sp (mm^−1^)	0.49 ± 0.02 ^a^	0.33 ± 0.07 ^b^	0.35 ± 0.06 ^b^	0.39 ± 0.04 ^b^	0.35 ± 0.04 ^b^	<0.05
Tb.N (mm^−1^)	0.54 ± 0.4 ^a^	1.93 ± 0.52 ^b^	2.16 ± 0.89 ^b^	1.89 ± 0.49 ^b^	2.21 ± 0.53 ^b^	<0.05
Conn.Dn (1/mm^3^)	0.000037 ± 0.000029 ^a^	0.000164 ± 0.000066 ^b^	0.000209 ± 0.000089 ^b^	0.000140 ± 0.000065 ^b^	0.000218 ± 0.000031 ^b^	<0.05
RPV (%)	-	6.82 ± 2.94 ^a^	5.75 ± 2.55 ^a^	4.49 ± 1.75 ^a^	5.56 ± 0.71 ^a^	>0.05

Data are presented as mean ± standard deviation (SD). Different superscript letters indicate significant differences between groups according to Tukey’s post hoc test. The *p*-value represents the overall between-group comparison by one-way ANOVA (*p* < 0.05).

**Table 3:** Percentage of red area and green area in groups C, XEN, XEN + L-PRF, XEN + A-PRF, and XEN + Bio-PRF.

Variable	C (Mean ± SD)	XEN (Mean ± SD)	XEN + L-PRF (Mean ± SD)	XEN + A-PRF (Mean ± SD)	XEN + Bio-PRF (Mean ± SD)	*p*-Value
Red Area (%)	1.85 ± 2.35 ^a^	1.92 ± 0.62 ^a^	3.72 ± 0.78 ^ab^	5.42 ± 0.95 ^b^	6.08 ± 1.15 ^b^	<0.05
Green Area (%)	12.32 ± 4.55 ^a^	13.2 ± 0.82 ^a^	6.8 ± 0.68 ^b^	4.2 ± 0.55 ^b^	5.0 ± 0.72 ^b^	<0.05

Data are presented as mean ± standard deviation (SD). Different superscript letters indicate significant differences between groups according to Tukey’s post hoc test. The *p*-value represents the overall between-group comparison by one-way ANOVA (*p* < 0.05).

## Data Availability

The datasets used and/or analyzed during the current study are available from the corresponding author on reasonable request.

## Abbreviations

The following abbreviations are used in this manuscript:

A-PRF	Advanced Platelet-Rich Fibrin
APCs	Autologous Platelet Concentrates
Bio-PRF	Horizontal Platelet-Rich Fibrin (Bio-PRF)
BP	Bone Porosity within the Volume of Interest (VOI)
BV/TV	Bone Tissue (Bone Volume/Total Volume)
CI	Confidence Interval
CSDs	Critical-Size Defects
Conn.Dn	Connectivity Density
EDTA	Ethylenediaminetetraacetic Acid
FGF	Fibroblast Growth Factor
HE	Hematoxylin and Eosin
IGF-1	Insulin-Like Growth Factor
L-PRF	Leukocyte- and Platelet-Rich Fibrin
MD	Mean Difference
Micro-CT	Micro-Computed Tomography
MSCs	Mesenchymal Stem Cells
NBA	New Bone Area
PDGF	Platelet-Derived Growth Factor
RPA	Remaining Particles Area
RP	Remaining Particles Volume
rpm	Rotations Per Minute
ROI	Region of Interest
TA	Total Area
Tb.N	Number of Trabecular Bones
Tb.Sp	Separation of Trabeculae
TGF-β	Transforming Growth Factor-Beta
VEGF	Vascular Endothelial Growth Factor
VOI	Volume of Interest
XEN	Bovine-Derived Xenogeneic Bone Grafts
